# Characteristic Analysis from Excessive to Deficient Syndromes in Hepatocarcinoma Underlying miRNA Array Data

**DOI:** 10.1155/2013/324636

**Published:** 2013-12-08

**Authors:** Qi-Long Chen, Yi-Yu Lu, Gui-Biao Zhang, Ya-Nan Song, Qian-Mei Zhou, Hui Zhang, Wei Zhang, Xin-sheng Tang, Shi-Bing Su

**Affiliations:** ^1^Research Center for TCM Complexity System, Shanghai University of TCM, Shanghai 201203, China; ^2^College of Life and Environment Science, Huangshan University, Huangshan 245021, China; ^3^Shanghai Longhua Hospital, Shanghai University of TCM, Shanghai 200126, China

## Abstract

Traditional Chinese medicine (TCM) treatment is regarded as a safe and effective method for many diseases. In this study, the characteristics among excessive, excessive-deficient, and deficient syndromes of Hepatocellular carcinoma (HCC) were studied using miRNA array data. We first calculated the differentially expressed miRNAs based on random module *t*-test and classified three TCM syndromes of HCC using SVM method. Then, the weighted miRNA-target networks were constructed for different TCM syndromes using predicted miRNA targets. Subsequently, the prioritized target genes of upexpression network of TCM syndromes were analyzed using DAVID online analysis. The results showed that there are distinctly different hierarchical cluster and network structure of TCM syndromes in HCC, but the excessive-deficient combination syndrome is extrinsically close to deficient syndrome. GO and pathway analysis revealed that the molecular mechanisms of excessive-deficient and deficient syndromes of HCC are more complex than excessive syndrome. Furthermore, although excessive-deficient and deficient syndromes have similar complex mechanisms, excessive-deficient syndrome is more involved than deficient syndrome in development of cancer process. This study suggested that miRNAs might be important mediators involved in the changing process from excessive to deficient syndromes and could be potential molecular markers for the diagnosis of TCM syndromes in HCC.

## 1. Introduction

Hepatocellular carcinoma (HCC) is one of the most common devastating cancer types, ranking sixth in incidence, and the third leading cause of cancer related deaths worldwide [1, 2]. The hepatitis B and hepatitis C infections, idiopathic cirrhosis, and alcoholic liver diseases usually are major risk factors for HCC [3]. For early diagnosis of HCC is difficult, and more than half of the HCC patients are diagnosed too late to benefit from the curative therapies. Furthermore, molecular approaches have revealed many molecular events associated with HCC, yet molecular mechanism of cell proliferation and effective biological markers for driving hepatocarcinogenesis remain largely unknown [4].

Under these circumstances, many failing patients seek to get help from complementary and alternative medicine, especially traditional Chinese medicine (TCM). TCM is an ancient Chinese medicine that evolved through at least three thousand years of uninterrupted clinical practice. The TCM treatment usually uses a traditional diagnosis method to classify the TCM syndromes, which based on clinical symptoms and signs, followed by the use of individualized treatment [5, 6]. For instance, Li et al. established a network balance model to evaluate the imbalanced network underlying TCM syndrome for gastritis patients [7, 8], which demonstrated that cold syndrome patients experience low levels of energy metabolism and immune regulation is intensified in hot syndrome patients.

MicroRNAs (miRNAs) are endogenous, noncoding, single-stranded small RNA molecules of approximately 22 nucleotides, which function as negative regulators that involve posttranscriptional gene expression through binding to the 3′-untranslated regions (3′-UTRs) of target mRNAs and consequently lead to mRNA cleavage or translational repression [9–11]. Although miRNAs are stable in circulation systems, tissue, and organ [12], they often can be explored in blood under pathological conditions, such as cell turnover and destruction, and pathological injury [13]. In the previous studies, we reported that circulating miR-583 and miR-663 refer to TCM syndrome differentiation in chronic hepatitis B [14] and the progression from excessive to deficient syndromes in chronic hepatitis B using miRNA-target dynamical network [15]. The results implicated that miRNAs are important mediators for TCM syndrome classification as well as CHB development progression and therefore could be potential diagnosis and therapeutic molecular markers.

In this work, we hypothesized that miRNAs expression levels and their target gene expression networks are the important factors for the TCM syndrome classification and the changes from excessive to deficient syndromes in HCC patients. We thus focused on the comparative analysis of the differences and similarities in the three TCM syndromes including liver-gallbladder dampness heat syndrome (LGDHS), liver depression and spleen deficiency syndrome (LDSDS), and liver-kidney yin deficiency syndrome (LKYDS). The aim is to demonstrate the change process of molecular mechanism from excessive syndrome to deficient syndrome at a network level by an integrative and comparative analysis of weighted miRNA-target network in HCC patients.

## 2. Materials and Methods

### 2.1. Clinical Specimens

In this work, clinical serum of 9 HCC patients and 7 healthy donors (Normal) were collected, whose were come from Shanghai Longhua Hospital. Then, these serums were subjected to miRNA microarray analysis. The diagnostic criteria of western medicine for HCC followed the guidelines defined by the Chinese Society of Hepatology and Chinese Society of Infectious Diseases in 2005 [16]. The TCM syndrome system for HCC applied by the 3 senior TCM doctors of each diagnosis was accepted according to the standards of TCM differential syndromes of viral hepatitis defined by the Internal Medicine Hepatopathy Committee of Chinese Traditional Medicine Association in 1991 [17]. This research project was approved by the local ethics committee of Shanghai University of TCM, and all patients were informed and consented for this study.

The differentiation of TCM syndromes in HCC patients was shown in Table 1. There were 3 LGDHSs, 3 LDSDSs, and 3 LKYDSs. In addition, 7 serums of normal control were randomly obtained from 120 individuals who had physically examination at Shanghai Longhua Hospital.

### 2.2. Serum Sample Collection and RNA Isolation

All serum samples were from the peripheral venous blood of HCC patients and healthy donors, which were immediately frozen in liquid nitrogen and then stored at −80°C. The RNAs in serum were extracted using a miRVana PARIS kit (Ambion, Austin, TX, USA) according to the manufacturer's protocol and using the RNase-free DNase (Promega, Madison, WI, USA) to eliminate DNA contamination. The concentration of RNAs isolated from serum ranged from 1.5 to 12 ng/*μ*L.

### 2.3. miRNA Microarray and Data Analysis

The profiles of serum miRNAs of 9 HCC patients and 7 normal controls were generated using Agilent Human miRNA microarray V3 (Agilent Technologies Inc, Santa Clara, CA, USA); 60 ng of RNA was labeled and hybridized for each array. Hybridization signals were detected with the Agilent microarray scanner; the data were extracted using Feature Extraction V10.7 (Agilent Technologies, CA, USA). All raw data were transformed into log 2 scale, and then, the expression levels were normalized by having zero mean and unit sample variance.

In order to evaluate the diversity of three TCM syndromes in HCC, we compared the miRNAs expressions of LGDHS, LDSDS, and LKYDS to normal, respectively. The relative miRNA expression levels were further normalized utilizing the median over the all patients, which make the each patient have a median log ration of 0 in normalized expression levels. The weighted differences miRNAs between TCM syndromes were calculated using the random variance model *t*-test and the fold-change >1.5 and *P* < 0.05 were considered significant. Heat-map analysis and hierarchical cluster analysis of expression data were performed using Cluster 3.0 and TreeView programs (the clustering calculation uses one minus correlation metrics and average linkages). Class prediction was performled using a statistical algorithm of the support vector machine (SVM) incorporating miRNA differentially expressed at a univariate parametric significance level of *P* = 0.01. The prediction rate was estimated via 10 fold and 10 times cross-validation and the bootstrap method for small sample data.

### 2.4. Identification and Prediction of miRNA Target Genes

Validated miRNA target genes were selected based on TarBase 6.0, which hosts the largest collection of manually curate experimentally validated miRNA-gene interactions [18]. Furthermore, the unverified miRNA target genes were predicted to regulating by miRNAs based on 10 programs, including DIANAmT, miRanda, miRDB, miRWalk, RNAhybrid, PicTar4, PicTar5, PITA, RNA22, and TargetScan. In these programs, the miRDB is different from others, which was using SVM learning machine to predict miRNA targets [19]. In order to increase the accuracy of predicted targets, we further screened prediction hits from two ways, that is, (i) random selected two sets from 9 programs (excepted miRDB) and intersected them, respectively, then united these intersection data as Data A; (ii) selected the miRDB data whose score >60, defined this data is Data B. Finally, the intersection between Data A and Data B acts as final data to build miRNA-target network.

### 2.5. Enrichment Analysis of Target Genes

Of the inferred miRNA target genes, those showing a significant (*P* < 0.05) expression difference between normal, LGDHS, LDSDS, and LKYDS samples were analyzed for pathways involving these genes using DAVID online analysis [20, 21], and significance analysis was determined when *P* values were corrected for false discovery rate (FDR). Gene sets containing less than 5 genes overlapping were removed from the DAVID analysis. In our analysis, GO terms and pathways with an FDR-adjusted *P* value of less than 0.05 were retained.

### 2.6. Weighted miRNA-Target Network Construction

We built the weighted miRNA-target gene networks for different syndromes of HCC by computing the miRNA and target gene degree distribution based on experimental validated target genes and predicted target genes, thus inferring the miRNA-target network in 3 TCMsyndromes of HCC, respectively. In the process of network building, miRNA nodes were weighted by their expression fold changes (absolute value of log⁡2), while target genes were weighted based on degree distributions between consecutive groups, and thus we obtained a node-weighted miRNA-target interaction network for each stage. In order to validate the veracity of above network, rank all nodes (miRNAs and target genes) of network according to their weights and test the similarity between them [15, 22]; thereafter, obtain deregulated nodes for mapping the network of consecutive TCM syndromes progression. In the weighted miRNA-target network, the nodes represent miRNAs or genes, and the edges represent the connection strength (adjacency).

## 3. Results and Discussion

### 3.1. Differential Expressed miRNAs of TCM Syndromes in HCC

**Figure 1:**
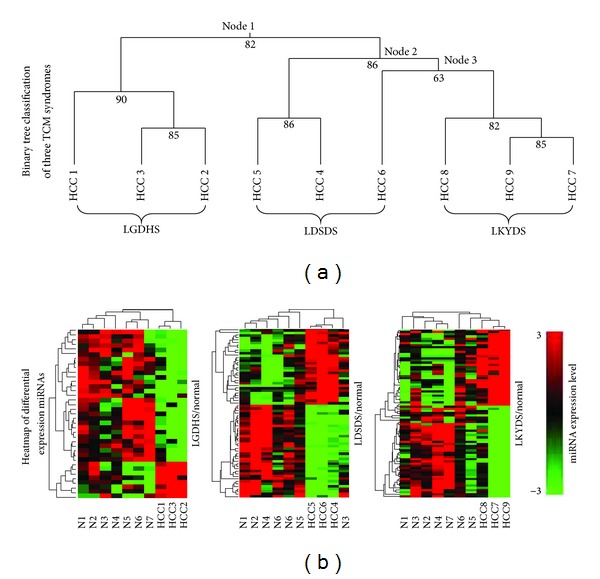
Cluster analysis of differential expression miRNAs in TCM syndromes in HCC. (a) Relationship among three typical syndromes of HCC divided by binary tree classification. (b) Heatmap of differential expressed miRNAs among the LGDHS/normal, LDSDS/normal, and LKYDS/normal.

The expressions of miRNAs were calculated and analyzed with random module *t*-test of R package, to search whether there are some significantly differential expressed miRNAs among the consecutive stages form excessive syndromes to deficient syndromes. 35 miRNAs in LGDHS/normal, 61 miRNAs in LDSDS/normal, and 71 miRNAs in LKYDS/normal differentially expressed. Three TCM syndromes of HCC were classified using a supervised learning algorithm (binary tree classification), and SVM act as prediction method. As shown in Figure 1(a), the excessive syndrome (LGDHS) was clearly classified to excessive-deficient combination syndrome (LDSDS) and deficient syndrome (LKYDS) (node 1, score = 82), which implicated that the excessive syndrome was distinctly different for other syndromes in HCC patients. However, we notice that the LDSDS samples first classified two classifications (node 2, score = 86) and then form a parallel branch cluster to LKYDS (node 3, score = 63). Although the topological profiles between LDSDS and LKYDS is clear-cut (Figure 1(a)), the lower SVM score (node 3, score = 63) shows the classification of them is still weakly. This result suggests the relationship between Excessive-deficient combination syndrome and Deficient syndrome is extrinsically closely in HCC patients.

Hierarchical cluster analysis revealed that the expression profiles of the differential miRNAs from each TCM syndrome were roughly classified, respectively. The consecutive heatmaps of differentially expressed miRNAs were shown in Figure 1(b). With the heat-maps, the miRNAs expression profiles of three syndromes were great differences, especially the Excessive syndrome (LGDHS/Normal) has great diversity to other syndromes. It implicates that the mechanism is individual for different typical syndromes of HCC. Interestingly, the hierarchical analysis shows that three LDSDS samples and one normal control (N3) first form a parallel branch and then cluster to other normal samples. This characteristic also was corresponding with the above profiles of LDSDS classification (Figure 1(a)). Because excessive-deficient combination syndrome (LDSDS) is a complicated TCM syndrome that includes both excessive syndrome and deficient syndrome [15], we infer that the LDSDS syndrome might have two features, which is compatible for excessive syndrome and deficient syndrome, and the miRNA-regulated mechanism is more complex than other syndromes in HCC.

### 3.2. Overview of the miRNA-Target Networks and Network Connections

**Figure 2:**
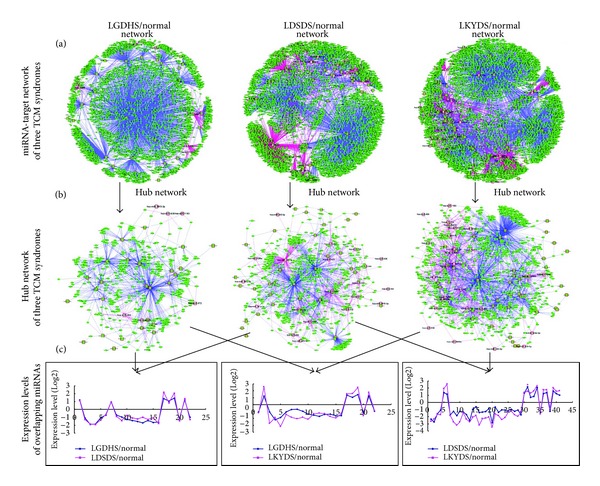
The miRNA-target networks and overlapping miRNA expression in TCM syndromes in HCC. (a) The global profiles of miRNA-target networks in three HCC TCM syndromes. (b) The hub networks of three syndromes from excessive to deficient syndromes. (c) The overlapping miRNA expression levels from the networks of three TCM syndromes.

As a gene regulator, a given miRNA usually has multiple different mRNA targets, and multiple miRNAs might target one gene [23]. In this study, using the validation database (Tarbase 6.0) and 10 predicted programs, miRNA target genes of three TCM syndromes were predicted, respectively. The final predictions were obtained by significant differences (*P* < 0.05) in each prediction program. Following the differential expressed miRNAs among LGDHS, LDSDS, and LKYDS in HCC, miRNA-target network for each syndrome was reconstructed based on predicted data. The global profiles of networks were shown in Figure 2(a). Noticeably, the topological profiles are more likely closed to “medusa” architecture [24], which consists of a regulatory core of hub nodes represented most prominently by miRNAs and target genes in network. It implies that the hub nodes (miRNAs or target genes) are much stronger determinants of the realized gene expression profiles, whereas the periphery nodes that should be regulated are not regulating. Furthermore, it also implicates the potential modules are subsistent in the networks, which in biological networks often represent molecular complexes and pathways [22].

To reveal the details of the regulatory core of network, the simplified network was reconstructed through the selected hub nodes. In this work, we defined a hub node that has more than 5 interactions in those TCM syndrome-specific networks, which represent these hub nodes might have highly effects for the topological structure of network. The reconstructed networks were shown in Figure 2(b), including the excessive syndrome (LGDHS/normal) network that consists of 35 miRNAs, excessive-deficient syndrome (LDSDS/normal) network that consists of 61 miRNAs, and Deficient syndromes (LKYDS/normal) network that consists of 71 miRNAs. Furthermore, there were 22 coexpression miRNAs between LGDHS/normal and LDSDS/normal, including 6 upexpression miRNAs. 41 miRNAs overlapped between LDSDS/normal and LKYDS/normal, and 5 miRNAs were upexpression; subsequently, LGDHS/normal shared 22 miRNAs with LKYDS/normal network, and including 12 up-expression miRNAs. The expression levels of overlapping miRNAs were shown in Figure 2(c). Although these overlapping miRNAs were appearance in three TCM syndromes, the difference of expression levels still indicated that they might play different roles form excessive to deficient syndromes in HCC. On the other side, the poor overlapping upexpression miRNAs also show a dramatic difference of deregulation in TCM syndromes, which suggested that LGDHS, LDSDS, and LKYDS have different molecular mechanisms.

### 3.3. Networks Prioritized Target Genes and Pathways in TCM Syndromes Progression in HCC

**Figure 3:**
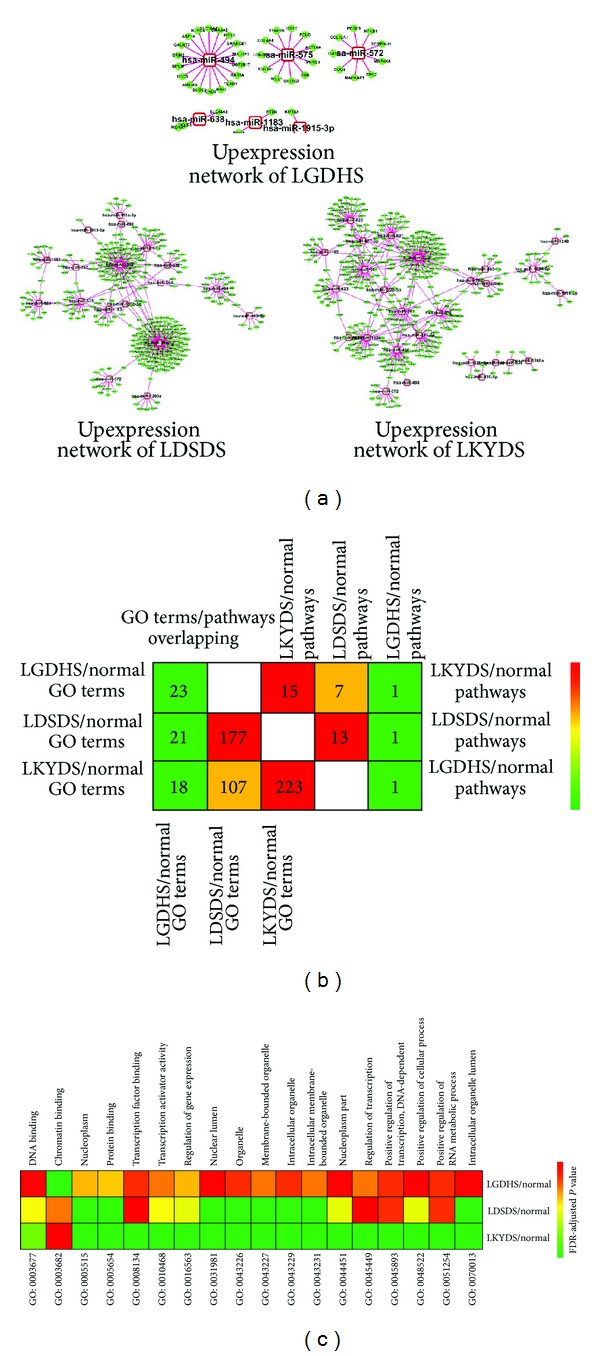
Upexpression miRNA-target networks, GO, and pathway terms in TCM syndromes progression in HCC. (a) Upexpression miRNA-target networks of LGDHS, LDSDS, and LKYDS in HCC. (b) Comparison of GO/pathway terms among the three networks of TCM syndromes. (c) The distribution of overlapping GO terms among the three networks of TCM syndromes. Numbers in the cell represent the number of overlapped GO terms in two networks. Colors were scaled according to the proportion of overlaps.

Because upexpression miRNAs might play roles that are more important in biological process, we divided the up-expression miRNA-target networks from the holistic networks for each TCM syndrome.  Figure 3(a) shows the up-expression networks (degree ≥5). We note that the proportion of up-expression miRNAs in LGDHS/normal is 18.8%, which was stringently less than LDSDS/normal (37.0%) and LKYDS/normal (39.7%). This great difference suggests that the molecular mechanism of excessive-deficient syndrome and deficient syndromes is more complex than excessive syndrome for HHC patients. Actually, these hub up-expression miRNAs usually affected the core cellular functions, such as immune responses and cell cycle in the molecular network via inhabited or degraded target genes [15]; furthermore, it also provides a new approach to distinguish the functional processes in disease progression [25, 26].

Because miRNA general inhibit translation or induce mRNA degradation by binding to the 3′-UTRs of target mRNAs [11]; here, we focus to conduct the GO terms and pathways analysis for the target genes of up-expression miRNAs using DAVID analysis [20, 21]. Significant analysis was determined when *P* values were corrected for false discovery rate (FDR). Gene sets containing greater than 5 genes overlapping were retained from the DAVID analysis. Analysis results with an FDR-adjusted *P* value of less than 0.05 were retained.

The representative GO and pathways terms of target genes in each TCM syndrome were shown in Figure 3(b). With the GO analysis, 21 GO terms were overlapping between LGDHS/normal and LDSDS/normal; 18 GO terms are overlapping between LGDHS/normal and LKYDS/normal, while LDSDS/normal and LKYDS/normal have 107 GO terms in common. In order to understand the GO terms holistically, 18 overlapped GO terms among LGDHS, LDSDS, and LKYDS were depth selected from original results based on *P* value and FDR less than 0.05. The distribution of overlapping GO terms was shown in Figure 3(c). Although these overlapping GO terms were appearance, actually, the FDR-adjusted *P* value of each GO term is different. For instance, the *P* values of GO: 0003677 (DNA binding), GO: 0031981 (nuclear lumen), GO: 0044451 (nucleoplasm part), GO: 0048522 (positive regulation of cellular process) and GO: 0070013 (intracellular organelle lumen) in LKYDS are clearly less than LGDHS or LDSDS. These results implicated that these GO terms are more stringently associated with deficient syndrome (LKYDS) and could deregulate the core cellular functions.

The pathway terms (KEGG and BIOCARTA) of target genes were also calculated using DAVID analysis. As shown in Figure 3(b), LKYDS/normal has 15 pathways, LGDHS/normal has 13 pathways, and LGDHS/normal has only 1 pathway. In addition, LGDHS/normal and LDSDS/normal, LGDHS/normal and LKYDS/normal only have one overlapped pathway, respectively, but LDSDS/normal and LKYDS/normal had 7 overlapped pathways. Obviously, this phenomenon suggested the mechanism of Excessive syndrome is different to other syndromes in HCC patients.

The detail of each pathway of three TCM syndromes was represented in Table 2. Compared with KEGG pathway, LGDHS is only related to small cell lung cancer, LDSDS is mainly associated with cell cycle, focal adhesion, endocytosis, and cancer pathways, and apoptosis, chronic/acute myeloid leukemia, and many cancer related pathways are associated with LKYDS. Compared with BIOCARTA pathway, the Deficient syndrome (LKYDS) is mainly associated with transcriptional regulation and map kinase pathways, while excessive-deficient syndrome (LDSDS) was more likely related to cell cycle (G1/S check point), cyclins, and cell cycle regulation. Previous researches have supported that imbalance of G1/S and G2/M phases is associated with dysfunction in hepatocarcinoma [27]. The results implicated that although excessive-deficient and deficient syndromes have similar complex mechanism, excessive-deficient syndrome have more dangers than deficient syndrome involved in the development of cancer process.

## 4. Conclusion 

In this study, based on miRNA microarray date of HCC patients and normal controls, we obtained evidence that the miRNA expression profiles of LGDHS, LDSDS and LKYDS are different. The classification results show that the Deficient syndrome (LKYDS) first cluster to Excessive-deficient syndrome (LDSDS), then as a parallel branch cluster to Excessive syndrome (LGDHS). Furthermore, the lower SVM score between LDSDS and LKYDS also suggested that the excessive-deficient syndrome is extrinsically close to deficient syndrome in HCC patients. The topological structure showed that the hub nodes (miRNAs or target genes) of miRNA-target network are much stronger determinants of the realized gene expression profiles, whereas the periphery nodes that should be regulated are not regulating. Obviously, the different topological profiles of networks also involved in the molecular mechanisms are different form Excessive to Deficient syndromes in HCC. GO and pathway analysis of target genes in up-expression networks revealed that the excessive-deficient and deficient syndromes of HCC are more complexity than excessive syndrome. Furthermore, although excessive-deficient and deficient syndromes have similar complex mechanism, excessive-deficient syndrome might have more dangers than deficient syndrome involved in development of cancer process.

## Figures and Tables

**Table 1:** Differentiation of TCM syndromes in HCC patients.

Patient ID	Age	Gender	TCM syndromes	TCM syndrome types
HCC 1	47	M	LGDHS	Excessive
HCC 2	63	M	LGDHS	Excessive
HCC 3	61	F	LGDHS	Excessive
HCC 4	62	M	LDSDS	Excessive-deficient
HCC 5	59	M	LDSDS	Excessive-deficient
HCC 6	58	M	LDSDS	Excessive-deficient
HCC 7	54	F	LKYDS	Deficient
HCC 8	52	M	LKYDS	Deficient
HCC 9	42	M	LKYDS	Deficient

**Table 2:** KEGG and BIOCARTA terms distribution of downregulated genes (upexpression level of miRNAs) of TCM syndromes in HCC.

Category	Term	*P* value	FDR value
LGDHS			
KEGG	Small cell lung cancer	0.0159	0.0141
LDSDS			
KEGG	Pathways in cancer	0.0054	0.0022
KEGG	Pancreatic cancer	0.0142	0.0043
KEGG	Glioma	0.0015	0.0157
KEGG	Prostate cancer	0.0036	0.0171
KEGG	Small cell lung cancer	0.0013	0.0313
KEGG	Non-small cell lung cancer	0.0224	0.0113
KEGG	Cell cycle	0.0017	0.0156
KEGG	Endocytosis	0.0110	0.0233
KEGG	Focal adhesion	0.0476	0.0436
BIOCARTA	Influence of Ras and Rho proteins on G1 to S transition	0.0287	0.0144
BIOCARTA	Regulation of BAD phosphorylation	0.0131	0.0291
BIOCARTA	Cyclins and cell cycle regulation	0.0321	0.0323
BIOCARTA	Cell cycle:G1/S check point	0.0471	0.0437
LKYDS			
KEGG	Prostate cancer	0.0048	0.0001
KEGG	Pathways in cancer	0.0001	0.0005
KEGG	Glioma	0.0001	0.0011
KEGG	Small cell lung cancer	0.0015	0.0013
KEGG	Melanoma	0.0019	0.0025
KEGG	Non-small-cell lung cancer	0.0020	0.0021
KEGG	Pancreatic cancer	0.0021	0.0022
KEGG	Chronic myeloid leukemia	0.0072	0.0813
KEGG	Apoptosis	0.0145	0.0163
KEGG	Acute myeloid leukemia	0.0436	0.0413
BIOCARTA	Y branching of actin filaments	0.0066	0.0771
BIOCARTA	NFAT and hypertrophy of the heart (transcription in the broken heart)	0.0108	0.0122
BIOCARTA	Influence of Ras and Rho proteins on G1 to S transition	0.0108	0.0122
BIOCARTA	Human cytomegalovirus and map kinase pathways	0.0136	0.0151
BIOCARTA	Overview of telomerase RNA component gene hTerc transcriptional regulation	0.0257	0.0268
